# Transgenerational effects of lambda-cyhalothrin on *Musca domestica* L. (Diptera: Muscidae)

**DOI:** 10.1038/s41598-022-23492-3

**Published:** 2022-11-10

**Authors:** Hamed A. Ghramh, Nauman Sadiq, Muhammad Nadir Naqqash, Allah Ditta Abid, Sohail Shahzad, Shafqat Saeed, Naeem Iqbal, Khalid Ali Khan

**Affiliations:** 1 Research Center for Advanced Materials Science (RCAMS), King Khalid University, P. O. Box 9004, Abha, 61413 Saudi Arabia; 2 Unit of Bee Research and Honey Production, King Khalid University, P. O. Box 9004, Abha, 61413 Saudi Arabia; 3 Biology Department, Faculty of Science, King Khalid University, P. O. Box 9004, 61413 Abha, Saudi Arabia; 4 Institute of Plant Protection, Muhammad Nawaz Shareef University of Agriculture, Multan, 60000 Pakistan; 5 Department of Plant Protection, Ministry of National Food Security and Research, Islamabad, Pakistan; 6 Applied College, King Khalid University, P. O. Box 9004, Abha, 61413 Saudi Arabia

**Keywords:** Entomology, Environmental impact

## Abstract

The hormetic effect may cause disease control measures to fail due to inadequate treatment of human disease vectors such as houseflies. Age-stage, two-sex life table is used for accurate estimation of the hermetic impacts on insects as it allows to study sub-lethal or transgenerational effects. Pyrethroids insecticides are primarily used for the management of houseflies. This study used lambda-cyhalothrin (a pyrethroid insecticide) to quantify its transgenerational impacts on houseflies. Life table parameters of a progeny of adult houseflies exposed to LC_10_, LC_30,_ and LC_50_ of lambda-cyhalothrin were computed. Statistically higher fecundity (71.31 per female) was observed in control treatment, while it was the adults exposed to LC_50_ recorded the lowest progeny. Significantly higher values for intrinsic rate of growth (*r*), limiting rate of growth (*λ*), and net reproductive rate (*R*_*o*_) (0.16, 1.16, and 31.38 per day, respectively) were recorded for the control treatment of the study. Contrarily, lower values for *λ, R*_*o,*_ and *r* were (0.10, 1.10, and 9.24 per day, respectively) were noted in the LC_50_ treatment. Decreased population parameters suggest that lambda-cyhalothrin can be successfully used in indoor environments to control houseflies.

## Introduction

The housefly [*Musca domestica* L. (Muscidae: Diptera)], is a noxious insect-pest of both large farms and households globally^1^. It is a major source of many dangerous foodborne infections in human environments, which represent its synanthropic characteristics^2,3^. Insect behavior to retain pathogens in their alimentary tract, and contamination of various exterior body parts during regurgitation, defecation, or feeding leads to food contamination^4^. Houseflies significantly aid in the spread of cholera, salmonellosis, and other severe foodborne diseases^5^. In contrast to helminthic (roundworms, hookworms, pinworms, and tapeworms), bacterial (shigellosis, salmonellosis, cholera), and protozoan (amoebic dysentery) infections, houseflies are also engaged in rickettsial and viral disorders^6^.

Insecticides are the first choice to manage housefly in animal sheds and urban vicinities^7,8^. However, potential health and environmental risks are associated with indoor application of pesticides. It has been observed that insecticide-based baits are more effective and ecologically safe in insect-vector control efforts than residual sprays^9,10^. Pyrethroid insecticides like lambda-cyhalothrin are among the most popular options for the management of insect-vectors at domestic and commercial scale. However, residual sprays of pyrethroid insecticides are relatively less dangerous for humans, kids, animals, and the environment as compared to other insecticides since they present significant exposure risks^11,12^. The use of baits could be less harmful to humans and the environment compared to the residual sprays. However, sound studies are needed on the impacts associated with the bait candidates for the management of insect-vectors.

Traditional bioassays have been unable to provide comprehensive information on the long-term use of insecticides in the baits^8^. Transgenerational studies on the offspring of the individuals exposed to certain stressor allow for in-depth examination of insecticides as a potential insect control method^13,14^. The use of insecticides may hormetically prime insect pests to become significantly more resilient to extreme circumstances by retaining stressful events. Several researchers have indicated that using insecticides at their sublethal dosages could lead to pest outbreaks^15–17^. The evolution of insecticide resistance in the vectors is a major failure cause of disease control programs^18^. Therefore, studying the transgenerational effects is necessary for the evaluation of any insecticide against insect vectors like houseflies prior to use. Age-stage, two-sex life table dealing with the age differentiation is the best option for developing efficient management programs for urban insect pests. Age-stage, two-sex life table is an excellent tool for evaluating population parameters of any species exposed to various environmental conditions or stresses^19^.

The application of sublethal doses of the pyrethroid deltamethrin on resistant insect species could result in a stimulating effect that makes exposed individuals grow bigger, a feature that can be passed down through generations. However, the sublethal effect can significantly vary among different insect species^20^. This study evaluated the impacts of lambda-cyhalothrin on housefly populations using age-stage, two-sex life table. Life table was constructed on the progeny of exposed and un-exposed housefly populations for comparison.

## Materials and methods

### Insect rearing

The housefly population consisted of adults collected from low chemical zone in Multan, Punjab, Pakistan. Insect rearing cages (30 × 30 × 60 cm) were used for the rearing of adults. Adult food was a mixture of sugar and yeast (70:30). Cotton soaked in 20% sugar solution was provided as water source. The adults were reared under 16:8 (Light: Dark), photoperiod. Plastic cups (300 g capacity) half-filled with larval diet were used for egg laying. Larval diet consisted of wheat paste containing rice husk, wheat bran, sugar, milk powder, and yeast in the ratio of 40:10:3:3:1, according to Bell et al.^21^. A new batch of larval food was provided after 2–4 days.

### Insecticide and solutions

The commercial formulation of lambda-cyhalothrin (Karate® 2.5 EC Syngenta Pvt. Ltd. Pakistan) was purchased from local market in Multan, Pakistan. Stock solution (500 µg/ml) of lambda-cyhalothrin was prepared in 20% sugar solution. Different insecticidal concentrations were obtained by serial dilution of insecticides in distilled water from the stock solution.

### Screening test

Food-incorporated bioassay designed by Kristensen et al.^22^ was followed to evaluate the acute toxicity of lambda-cyhalothrin to housefly. Bioassay was conducted in 30 × 30 cm boxes. A mesh was installed on the sides of the boxes to ensure proper ventilation. The insecticide solutions of 1.56, 3.12, 6.25, 12.5, 25, and 50 µg/ml were used in the bioassays. A cotton plug was soaked in sugar or insecticide solution.

A total 30 individuals were exposed to a single treatment (10 in each replication with equal male and female ratio). Mortality was recorded 48 h after insecticide treatment to calculate LC_10_, LC_30_ and LC_50_ values, respectively. The housefly populations were reared for five consecutive generations prior to use in the experiments.

### Transgenerational studies

After computing LC_10_, LC_30_ and LC_50_ values, a total of 50 adults were exposed to calculated values of insecticide and an untreated control. After exposing the adults, larval medium was provided for egg laying (48 h after the treatment). Eggs of the exposed adults were randomly selected for transgenerational effects on population and demographic parameters.

Adults (F_1_) of houseflies were monitored daily to calculate the adult longevity and the values for fecundity were calculated. Newly hatched eggs were continuously removed and counted until the adult died in the treated population. The methodology was same for all the treatment and control to make precise comparison. The larval development, survival, and growth were recorded from hatching to adulthood. The data were recorded daily until the mortality of individuals. Transgenerational studies were conducted under 27 °C temperature, 65–75% relative humidity and of 12:12 (L: D) h photoperiod. The collected data from this experiment was used to construct age-stage, two-sex life table.

### Statistical analysis

The raw data on life table parameters from each exposed individual were analyzed according to Chi and Su^23^. Age stage two-sex life table theory was used to analyze the data for life table according to Chi and Liu^24^ and Chi^25^. The life table parameters, i.e., net productive rate, *S*_*xj*_ (age-stage specific survival rates), *T* (mean generation time), *l*_*x*_ (age-specific survival rate), *m*_*x*_ (age-specific fecundity), *r* (Intrinsic rate of increase), *e*_*xj*_ (age-stage specific life expectancy), TPOP (total pre-oviposition period), APOP (adult pre-oviposition period), and R (reproductive value) were computed. The intrinsic rate of growth was calculated by the following formula;

$$\sum\limits_{x = 0}^{\infty } {e^{ - r(x + 1)} } l_{x} m_{x} = 1$$

The formula to compute age stage-specific survival is given below. Using the same formula, value for *S*_*xj*_ was computed.

$$lx = \sum\limits_{j = 1}^{k} {S_{xj} }$$

The *R*_*o*_ was computed by using the following equation:

$$R_{0} = \sum\limits_{x = 0}^{\infty } {l_{x} m_{x} }$$

Similarly, the below equation was used to compute *T*.

$$T = \left( {{\text{In}}R_{o} } \right)/r$$

The value of r was estimated by using the formula given below:

$$\sum\limits_{x = 0}^{\infty } {e^{ - r(x + 1)} } l_{x} m_{x} = 1$$

The value of *λ* (finite rate of increase) was calculated as under*:*

$$\lambda = e^{r}$$

The *e*_*xj*_ was calculated according to the following equation:

$$e_{xj} = \sum\limits_{i = x}^{\infty } {\sum\limits_{y = j}^{k} {s_{iy}^{\prime } } }$$

The equation for estimating *v*_*xj*_ value is given below:

$$v_{xj} = \frac{{e^{ - r(x + 1)} }}{{S_{xj} }}\sum\limits_{i = x}^{\infty } {e^{ - r(x + 1)} } \sum\limits_{y = j}^{k} {s_{iy}^{\prime } f_{iy} }$$

TWO SEX- MSChart program was used to analyze various life table parameters. The 100,000 random resampling were performed using the bootstrap method to determine the variances in population parameters and standard errors^26^. The comparison was made between population parameters of control and insecticide-treated groups, and among generations of each treatment group. The confidence intervals of differences were generated by Two Sex-MSChart^27^.

## Results

### Lethal concentration estimation

The LC10, LC30 and LC50 values for Lambda-cyhalothrin were 41.22 μg/ml, 14.47 μg/ml and 0.25 μg/ml. Where the value of chi-square was calculated as 1.42 (Table 1).

**Table 1:** Toxicity of lambda-cyhalothrin to housefly adults after 48 h.

Insecticide	LC_10_ (95% FL) (µg mL^−1^)	LC_30_ (95% FL) (µg mL^−1^)	LC_50_ (95% FL) (µg mL^−1^)	χ^2^	DF	*P*
Lambda-cyhalothrin	0.25 (0.03–1.54)	14.46 (3.74- 24.06)	47.07 (29.90–83.26)	1.420	4	0.841

*LC* lethal concentration, *DF* degree of freedom, *χ*^2^ Chi square.

### Life table parameters

Life table parameters, i.e., oviposition, male longevity, APOP, TPOP, and fecundity are given in Table 2. Data from the control group and experimental groups were used to compare the parameters (under three different concentrations, i.e., LC_10_, LC_30_, and LC_50_). Pre-adult duration in F_1_ individuals treated with lambda-cyhalothrin was significantly reduced from 13.85 d (LC_50_) to 12.26 d (LC_10_), while the value for the pre-adult duration in control 13.12 d was lower than LC_30_ and LC_50_ (*P* < 0.0001). Female longevity of F_1_ was significantly higher in control (27.09 d), followed by LC_10_ (22.50 d), while significantly lower value (21.61 d) was noted for LC_30_.

**Table 2:** Transgenerational effect of Lambda-cyhalothrin on different life table parameters of *Musca domestica.*

Parameters	Control	LC_10_	LC_30_	LC_50_
Pre-adult duration (days)	13.12 ± 0.05a	12.26 ± 0.15a	13.81 ± 0.07a	13.85 ± 0.06a
Female longevity (days)	27.09 ± 0.54a	22.50 ± 0.64b	21.61 ± 0.91b	22.13 ± 1.08b
Male longevity (days)	24.8 ± 0.77a	22.14 ± 0.54a	21.68 ± 0.81a	23.61 ± 1.02a
APOP (days)	3.86 ± 0.07b	3.31 ± 0.11b	4.00 ± 0.00b	5.00 ± 0.00a
TPOP (days)	17.00 ± 0.00b	15.56 ± 0.13b	17.54 ± 0.16b	19.00 ± 0.00a
Oviposition days	8.09 ± 0.31a	4.12 ± 0.39b	3.72 ± 0.43b	4.23 ± 0.70b
Fecundity (per female)	71.31 ± 2.34a	34.00 ± 3.20b	36.76 ± 7.83b	30.80 ± 7.76b

*TPOP* total pre-oviposition period of female counted from birth, *TPOP* total pre-oviposition period of female counted from birth.

Means in the same row followed by the same letter are not significantly different (*P* > 0.05) using bootstrap test.

Male longevity was reduced from 24.8 days (control group) to 21.68 days (LC_30_). The APOP was significantly higher in the individuals exposed to LC_50_ (5.00 d), whereas lower values were recorded for LC_10_ (3.31 d) and control (3.86 d). The TPOP value was significantly higher (19.00 d) at LC_50_, followed by LC_30_ (17.54 d) and control (17.00 d), while significantly lower (15.56 d) values were recorded for LC_10_. Oviposition days were significantly lower in the population exposed to LC_30_ (3.72 days). Furthermore, the value for oviposition days was significantly higher in control treatment (8.09 days) compared to the rest of the treatments included in the study. The value was twice higher in control compared to LC_30_-exposed population. Fecundity was also higher in control population (71.31/female), whereas, the values were lower (30.80/female) in the population exposed to LC_50_.

### Population parameters

Sublethal doses of the insecticide were used to study various population parameters. The studied parameters included net reproductive rate (*R*_*o*_), finite rate of increase (*λ*), intrinsic rate of increase *(r*), and the mean generation time (*T*). These parameters are presented in Table 3. Significant variation was noted between insecticide-treated and control group in term of main population parameters*.* The *r* was higher in the control group (0.16/day), and lower in the LC_50_-exposed population (0.11/day). The *R*_*o*_ of control population (31.38/day) was significantly higher than the population exposed to LC_10_ (10.88/day). The *R*_*o*_ was significantly lower (9.24 days) in the population exposed to LC_50_. The *T* was significantly higher in the population exposed to LC_50_ (22.88/day), while lower values were noted for the population exposed LC_10_ (19.31/day). The *λ* significantly differed among insecticide treatments and control. The highest value was noted for control (1.17/day), and the lowest value for LC_50_ (1.10 per day).

**Table 3:** Transgenerational effect of Lambda cyhalothrin on different life table parameters of *Musca domestica.*

Parameters	Control	LC_10_	LC_30_	LC_50_
*r* (per day)	0.16 ± 0.01a	0.13 ± 0.01b	0.11 ± 0.02b	0.11 ± 0.01b
*R*_*o*_ (per day)	31.38 ± 5.12a	10.88 ± 2.46b	9.56 ± 3.01b	9.24 ± 3.03b
*T*	21.06 ± 0.10b	19.31 ± 0.27b	20.81 ± 0.20b	22.88 ± 0.52a
*λ* (per day)	1.17 ± 0.01a	1.14 ± 0.01b	1.11 ± 0.02b	1.12 ± 0.02b

*r* = The intrinsic rate of increase (per day); *ʎ* = The finite rate of increase (per day);

*r* = intrinsic rate of growth; *R*_*0*_ = The net reproductive rate (offspring/individual); *T* = The mean generation time (days), *λ* = limiting rate of growth.

Means in the same row followed by the same letter are not significantly different (*P* > 0.05) using bootstrap test.

### Age-stage specific survival rate (***s***_***xj***_)

**Figure 1:**
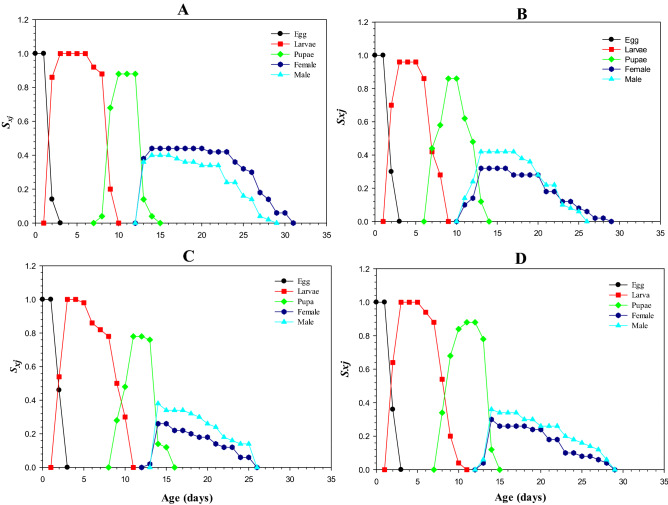
Transgenerational effect of lambda-cyhalothrin, (**A**) control, (**B**) LC_10_, (**C**) LC_30_, and (**D**) LC_50_ on Age stage specific survival rate (*S*_*xj*_) of *Musca domestica.*

The higher values of *S*_*xj*_ were recorded for males and females in control and LC_10_, which were around 0.44 (day 12 to 17) for males, and 0.32 (Day 12 to 16) for females (Fig. 1A and B). The peak *S*_*xj*_ values in male and female adults were 0.26 and 0.38 on 14th day in LC_30_ treatment (Fig. 1C). The *S*_*xj*_ in males declined to 0 on 25th day, while similar value for females was noted on 29th day. The variation and overlapping in *S*_*xj*_ curves indicated that individuals exposed to different concentrations develop at different rates. The lowest peak for *S*_*xj*_ was observed in LC_50_-exposed males (Fig. 1 D).

### Age-stage life expectancy (*e*_*xj*_)

**Figure 2:**
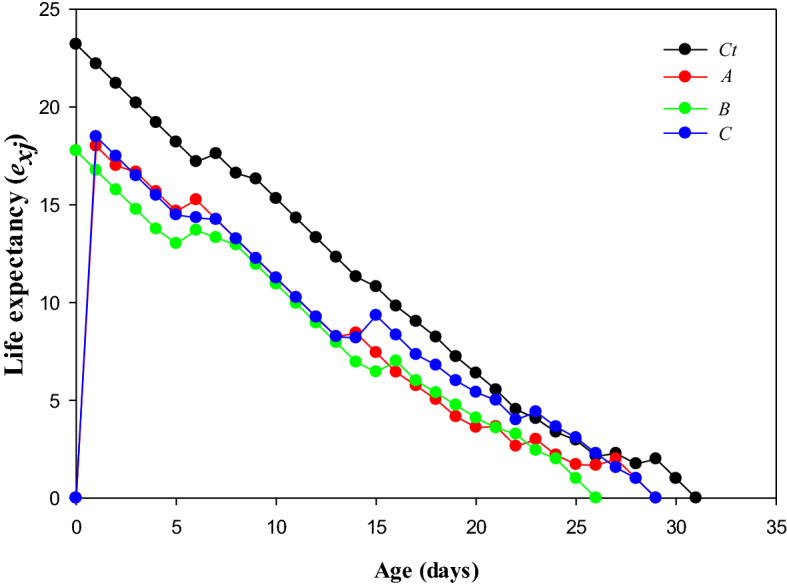
Transgenerational effect of lambda-cyhalothrin on Life expectancy *(e*_*xj*_*)* of *Musca domestica* *Ct) control, A) LC_10_, B) LC_30_, and C) LC_50_.

The *e*_*xj*_ is an indication of expected time length for an individual at stage *j* and age *x* (Fig. 2). The *e*_*xj*_ value was higher in control treatment (reached to 0 on 31st day), whereas adults treated with LC_50_ recorded lower value for *e*_*xj*_ (reached to 0 on 29th day).

### Age-specific maternity (***l***_***x***_***m***_***x***_)

**Figure 3:**
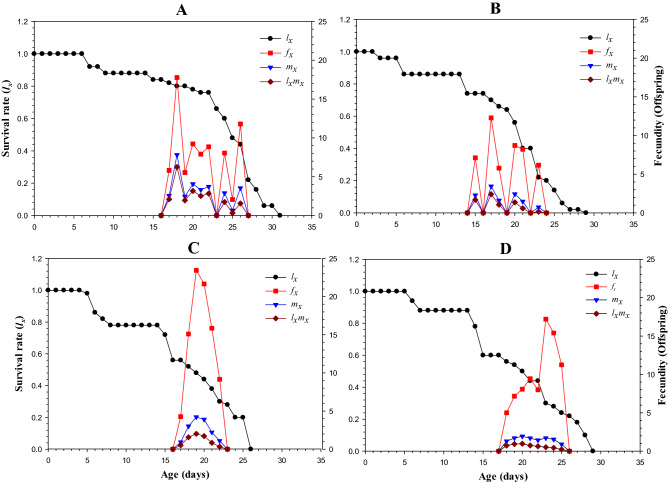
Transgenerational effect of Lambda-cyhalothrin, (**A**) control, (**B**) LC_10_, (**C**) LC_30_, and (**D**) LC_50_ on Age-specific survival rate (*l*_*x*_), age-specific fecundity of total population (*m*_*x*_), and age-specific maternity (*l*_*x*_*m*_*x*_) of initial *Musca domestica* exposed to LC_10_, LC_30_ and LC_50_ of lambda-cyhalothrin in F_5_.

Age-specific maternity is another important factor in determining population parameters. Age-specific maternity (*l*_*x*_*m*_*x*_) is a composite of two parameters, i.e., age-specific survival rate (*l*_*x*_) and the overall population's age-specific fecundity (*m*_*x*_). These cumulative parameters are shown in Fig. 3. The age-stage specific fecundity (*f*_*x*_) was significantly higher in progeny of LC_30_-treated adults (23.44 offspring at the age of 19 days), while the lowest value was noted for the progeny of the adults exposed to LC_10_ (12.37 offspring on day 17).

The age-stage specific survival (*l*_*x*_) gradually decreased and *l*_*x*_ value was lower in LC_30_ compared to other treatments of the study. The age-specific maternity (*l*_*x*_*m*_*x*_) was highest in the control (7.82 offspring at day 18) and lowest in LC_50_ treatment (1.94 offspring at day 20).

### Age-stage reproductive values (***V***_***xj***_)

**Figure 4:**
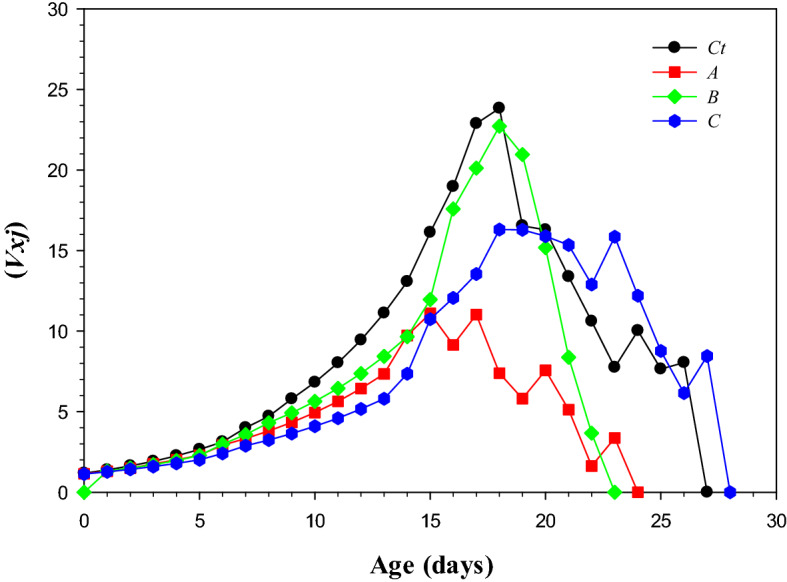
Transgenerational effect of lambda-cyhalothrin on reproductive value (*V*_*xj*_) on *Musca domestica* *Ct) control, A) LC_10_, B) LC_30_, and C) LC_50_.

Age-stage reproductive (*V*_*xj*_) is given in Fig. 4. The reproductive value curves clearly indicate the variation in data. The higher *V*_*xj*_ value (23.83 on day 18) was noted for the control treatment. The *V*_*xj*_ curve showed a clear decline in the value starting at 18th day 18, which declined to 0 on 27th day. The LC_10_ population had the lowest peak value (11.21 on 15th day) which decreased to 0 on 24th day.

## Discussion

The lambda-cyhalothrin has been reported to possess high toxicity against insects. The modes of action of the pesticide is ingestive, contact, and ovicidal. Lambda-cyhalothrin insecticide is frequently used for the management of housefly especially in indoor environment^28^. In this study, lambda-cyhalothrin demonstrated high and acute toxicity against adult houseflies after 48 h of food incorporation bioassay. In addition to lethal effects of the insecticides, insect populations are routinely exposed to sublethal doses of the applied insecticides in the field due to variability in pesticide distribution variability and continual degradation^29,30^.

Life tables are important tools to estimate age stage-specific survival, net reproductive rate, and growth of insects under varying conditions. Intrinsic rate of growth (*r*) is determined by birth rate minus death rate over time^31^. The value for *r* will assist in predicting the possible implications of insect population after the application of a chemical control strategy^25,32^. Results indicated that female longevity (27.09 days) and fecundity (71.31 eggs/female) were higher in the progeny of control treatment, which coincide with the findings of Sylvestre et al.^33^. Results of the current study are also close to the findings of other researchers who reported reduced survival in insects after exposure to insecticides^34,35^. Reduced fecundity in the progeny of insecticide-exposed populations was observed in this study. Similar results have been reported by Michaelides et al.^36^, and Laycock et al.^37^. Nevertheless, it is known that insect pests respond to stress with a variety of behaviors, including longer adult lifespan^38^.

Another important factor for population estimation is oviposition period. Of oviposition days; the data showed APOP and TPOP values as 3.86 and 17.00, respectively which are lower than control group (8.09 days).Ioriatti et al.^39^ also argued that fecundity was earlier and oviposition days were higher in the control group. This increase indicates that the populations can propagate efficiently in the absence of external stressors^40^. The results of the current study are also in accordance with the findings of Yao et al.^41^ who reported decreased egg-laying in *Conopomorpha sinensis* after insecticide exposure. Additionally, exposure to sublethal doses of insecticide decreased fecundity in *Chilo suppressalis* larvae and vitellogenin gene expression in female adults^42^.

Dolezal^43^ also reported reduced reproduction rate due to insecticides’ exposure in bees. The highest value (22.88 days) for *T* was noted for LC_50_. This increase in *T* under stress conditions is like the findings of Sial et al.^44^ who evaluated the effectiveness of lambda-cyhalothrin against *Myzus persicae* and reported an increase in mean generation time after exposure to sublethal doses of insecticides. Tan et al.^8^ also reported similar results for *T* in the population exposed to lambda-cyhalothrin. Moreover, *S*_*xj*_ was higher at LC_10_ on day 12. The increase in survival rate in green peach aphid has been reported by Wang et al.^45^. Contrarily, life expectancy (*e*_*xj*_) is an important parameter for population and survival estimation. In this study the lowest peak for *e*_*xj*_ was noted for LC_50_ which indicates that lambda-cyhalothrin could reduce expected survival. The higher values for expected survival in control and the lowest in LC_50_ are analogous to the results of Hatami et al.^46^. In this experiment, peak for age-specific maternity (*l*_*x*_*m*_*x*_) was higher in control compared to other treatments. These results are in accordance with the results of Marcic^47^, who reported that acaricides greatly contribute in reducing insect growth. The peak for age stage-specific reproductive value *(V*_*xj*_*)* was highest in control group and this peak can be justified by comparing with the results of Rimy et al.^48^. The higher *V*_*xj*_ peak in control can be limited efficiently by the usage of lambda-cyhalothrin. As a matter of fact, metabolic enzymes can alter the biological processes. Detoxification enzymes, such as cytochrome P450 and monooxygenases (P450) are known to metabolize insecticides^18,49^. Moreover, P450 enzymes are also involved in biosynthetic pathways related to insect growth and reproduction^50^. Though various control methods like CRISPR, RNAi and several formulations of biopesticides are gaining importance nowadays^51,52^. Additionally, they are ecofriendly too. However, chemical control is still preferred by community in developing and/or under-developed countries^18^. Also, RNAi does not work well in houseflies while further research work is required for choosing CRISPR as a control option for highly notorious pests like housefly found in a variety of ecological zones. Contrarily, biopesticides are slower in action and take time to control while in the case of household pests quick control is required^53,54^.

## Conclusion

Variation in the development rate is a common phenomenon in biology. If these variations are ignored, they may affect the fecundity and survival curves. The study of these changes is considered an important component in developing precise life table. The key benefits of two-sex life table analysis are that the developmental variability rates among different individuals are considered, rather than development times, total population is discussed during the analysis, including males, females, and those who die before reaching adulthood. Simulation studies are also thought to be extremely useful. Because of their different susceptibilities to insecticides, different stages are focused. The behavior pattern in different stages is also different. Furthermore, the study's main conclusion is that Pyrethroids, specifically lambda-cyhalothrin, is an effective insecticide for the control of housefly*.* It could be used as a component of traps or baits to control this pest. The parameters investigated during this experiment suggest that it could be a useful population suppressor.

## Supplementary Information

Supplementary Information 1.

Supplementary Information 2.

Supplementary Information 3.

Supplementary Information 4.

## Data Availability

All data generated or analyzed during this study are included in this published article as supplementary files: Control Population Data, LC10 Selected Population Data, LC30 Selected Population Data and LC50 Selected Population Data.
